# Mitochondrial Bioenergetics of Metastatic Breast Cancer Cells in Response to Dynamic Changes in Oxygen Tension: Effects of HIF-1α

**DOI:** 10.1371/journal.pone.0068348

**Published:** 2013-06-28

**Authors:** Anne R. Diers, Praveen K. Vayalil, Claudia R. Oliva, Corinne E. Griguer, Victor Darley-Usmar, Douglas R. Hurst, Danny R. Welch, Aimee Landar

**Affiliations:** 1 Department of Pathology, University of Alabama at Birmingham, Birmingham, Alabama, United States of America; 2 Department of Surgery, University of Alabama at Birmingham, Birmingham, Alabama, United States of America; 3 Center for Free Radical Biology, University of Alabama at Birmingham, Birmingham, Alabama, United States of America; 4 Comprehensive Cancer Center, University of Alabama at Birmingham, Birmingham, Alabama, United States of America; 5 Department of Cancer Biology, Kansas University Medical Center, Kansas City, Kansas, United States of America; University of Medicine and Dentistry of New Jersey, United States of America

## Abstract

Solid tumors are characterized by regions of low oxygen tension (OT), which play a central role in tumor progression and resistance to therapy. Low OT affects mitochondrial function and for the cells to survive, mitochondria must functionally adapt to low OT to maintain the cellular bioenergetics. In this study, a novel experimental approach was developed to examine the real-time bioenergetic changes in breast cancer cells (BCCs) during adaptation to OT (from 20% to <1% oxygen) using sensitive extracellular flux technology. Oxygen was gradually removed from the medium, and the bioenergetics of metastatic BCCs (MDA-MB-231 and MCF10CA clones) was compared with non-tumorigenic (MCF10A) cells. BCCs, but not MCF10A, rapidly responded to low OT by stabilizing HIF-1α and increasing HIF-1α responsive gene expression and glucose uptake. BCCs also increased extracellular acidification rate (ECAR), which was markedly lower in MCF10A. Interestingly, BCCs exhibited a biphasic response in basal respiration as the OT was reduced from 20% to <1%. The initial stimulation of oxygen consumption is found to be due to increased mitochondrial respiration. This effect was HIF-1α-dependent, as silencing HIF-1α abolished the biphasic response. During hypoxia and reoxygenation, BCCs also maintained oxygen consumption rates at specific OT; however, HIF-1α silenced BCC were less responsive to changes in OT. Our results suggest that HIF-1α provides a high degree of bioenergetic flexibility under different OT which may confer an adaptive advantage for BCC survival in the tumor microenvironment and during invasion and metastasis. This study thus provides direct evidence for the cross-talk between HIF-1α and mitochondria during adaptation to low OT by BCCs and may be useful in identifying novel therapeutic agents that target the bioenergetics of BCCs in response to low OT.

## Introduction

Partial pressure of oxygen (*pO_2_)* is a micro-environmental factor that plays key roles in tumor progression and responses to treatment. Limited oxygen delivery is known to occur in both solid tumors with aberrant microcirculation [1] and avascular microscopic tumors [2]. Two types of tumor cell hypoxia (defined as OT <1%) have been observed [3]. Chronic, prolonged hypoxia, also termed ‘diffusion-limited hypoxia,’ develops in regions distal from blood vessels. These areas of the tumor are characteristically adjacent to necrotic regions and occur as a consequence of permanent limitations in oxygen diffusion [4]. On the other hand, intermittent hypoxia results from acute and fluctuating changes in OT which may last for less than a minute to several hours in tumors. Intermittent hypoxia can develop as a result of temporary obstruction or cessation of tumor blood flow [5]. Exposure of tumor cells to either chronic or acute hypoxia initiates signaling events which promote spontaneous metastasis [6], [7]; however, some studies suggest that intermittent cycles of acute hypoxia are more significant in driving the metastatic process than chronic hypoxia [8], [9].

In response to low OT, cells rely on two general responses to avoid depleting oxygen levels to anoxia (0% oxygen) and maintain cellular energy status. These include increasing oxygen supply to the tissue and decreasing cellular oxygen demand [10]. The adaptive responses to low OT are orchestrated by a family of transcription factors which are responsive to loss of oxygen including hypoxia inducible factor-1 (HIF-1), early growth response-1 (Egr-1), activator protein-1 (AP-1), and nuclear factor-κB (NF-κB). Among these, the HIF-1 pathway is the most well characterized [11]. HIF-1 is a key regulator of cellular bioenergetic function, commonly working in conjunction with oncogene activation (e.g. c-MYC) and tumor suppressor loss/mutation (e.g., p53) to coordinate decreased mitochondrial oxidative phosphorylation (OXPHOS) and enhanced aerobic glycolysis. In fact, regulation of these metabolic pathways underpins the Warburg effect [12], [13]. Thus, oxygen-sensing and adaptation to low OT are critical for survival of cancer cells in various tumor microenvironments and progression to an increasingly malignant phenotype.

Studies have been performed to explore the effects of acute and chronic hypoxia on cellular respiration in multiple cell types including cardiomyocytes [14], hepatocytes [15]–[17], human and murine fibroblasts [18], and cancers of the breast [19], colon [18], [20] and kidney [18] carcinoma cells. In general, cells exposed to acute hypoxia (<30 min) did not exhibit a decrease in oxygen consumption rate (OCR). However, exposure to chronic hypoxia for longer periods (>2 h to 6 days) caused significantly suppressed mitochondrial oxygen consumption and increased lactate production [14], [18], [19]. In hepatocytes and cardiomyocytes, this effect appears to be mediated by decreased ATP utilization and regulation of complex IV of the mitochondrial respiratory chain [14]–[16], [21]. In cancer cells, however, decreased mitochondrial oxygen consumption after chronic hypoxia has been attributed to HIF-1-dependent metabolic reprogramming through specific transcriptional events [18], [22].

While effects of long-term exposure to low OT on cancer cell metabolism and bioenergetics have been extensively studied [3], [13], [18], [22], how cancer cells adapt their bioenergetics to various OTs in real-time is not well understood. This information is very important, as it reflects the bioenergetic behavior of cancer cells, an emerging hallmark of cancer [23], [24]. Moreover, these bioenergetic responses to different OTs are challenging to determine *in vivo.* Thus, information on the differences between normal and cancer cell bioenergetics under different OTs is critical and may be useful to develop therapeutic agents targeted to the cancer cell bioenergetics for personalized cancer therapy. In the present study, we clearly show using highly sensitive extracellular flux analysis that in contrast to non-tumorigenic breast epithelial cells, BCCs rapidly adapt to decreasing OT by enhancing glycolysis and altering mitochondrial function in a biphasic manner. This response is characterized by an increase in basal oxygen consumption mediated by HIF-1α which is attributable to increased activity of the mitochondrial respiratory chain, and a decrease in oxygen consumption which is due to lack of oxygen availability at lower OT.

## Materials and Methods

### Materials

All chemicals were of analytical grade and purchased from Sigma-Aldrich (St. Louis, MO) unless otherwise noted. 2-[N-(7-Nitrobenz-2-Oxa-1,3-Diazol-4-yl)Amino]-2-Deoxy-D-Glucose (2-NBDG) was purchased from Invitrogen (Carlsbad, CA).

### Cell Culture

MCF10A, an immortalized human breast epithelial cell line, was obtained from American Type Culture Collection (ATCC, Manassas, VA). MDA-MB-231 (MB231), MCF10CA clone a.1 (CA a.1), and MCF10CA clone d.1α (CA d.1α) human metastatic BCCs were described previously [25], [26]. MCF10A cells were maintained in a mammary epithelial cell culture medium (MEGM; 8 mM glucose) purchased from Lonza (Walkersville, MD). MB231 cells were cultured in RPMI 1640 (11 mM glucose) supplemented with 10% fetal bovine serum (FBS), and CA a.1 and CA d.1α cells were maintained in DMEM-F12 (17.5 mM glucose) supplemented with 5% FBS and non-essential amino acids.

### HIF-1α Silencing

HIF-1α silenced clones were prepared by transfection of plasmids carrying shRNAs specific for human HIF-1α. Plasmids were purchased from Open Biosystems (ATCC Integrated Molecular Analysis of Genomes and their Expression (IMAGE) catalog no. RHS4533-EG3091). To generate stable cell lines, 5 µg of TRCN0000010819, TRCN0000003811, TRCN0000003808, TRCN0000003810 and TRCN0000003809 or empty vector (pLKO.1) were transfected into MCF10CAd.1α and MB231cells with the FuGENE 6 transfection reagent. Two days after transfection, cells were selected with 2 µg/ml puromycin for 4 weeks. Clones were assayed for protein expression by immunoblotting with monoclonal antibodies against HIF-1α (BD Transduction Laboratories, #610958). Several positive clones were expanded, and clones with the lowest HIF-1alpha expression were chosen for further use in this study.

### Measurement of OCR and ECAR

For extracellular flux assays (XF assays), cells were seeded at a density of 4×10^4^ cells/well for MCF10A and MB231 cells, 3×10^4^ cells/well for CA a.1 and CA d.1α cells 24 h prior to the assay. XF assays were performed in the same aforementioned basal media without bicarbonate supplemented with 0.5% FBS and HEPES (5 mM), pH 7.4. The buffer capacity of each media was determined prior to the assay. Cells were equilibrated in a non-CO_2_ incubator for 1 h before measurement of oxygen consumption rates (OCRs) and extracellular acidification rates (ECARs). In order to determine the role of glycolysis in the XF assays, 2-deoxy-D-glucose (2DG; 20 mM) was added to the assay media; thus, cells were exposed to 2DG for 1 h prior to extracellular flux analysis.

### Measurement of OCR and ECAR during Adaptation to Low OT

**Figure 1:**
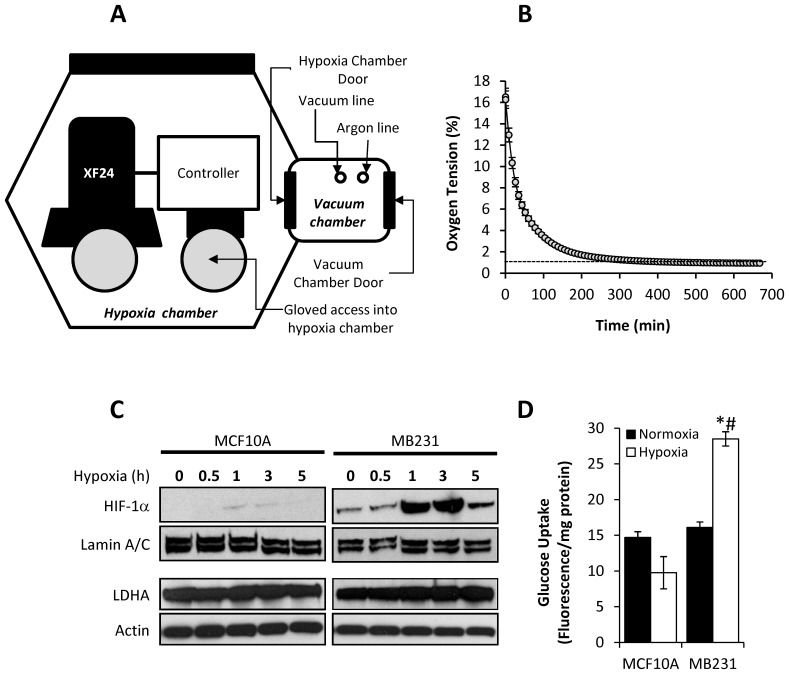
Validation of experimental setup to measure OCR and ECAR of breast epithelial and cancer cells under different OTs. A schematic representation of exposure to different OTs in the Seahorse XF24 Analyzer is shown (**A**). Representative O_2_ traces in media were obtained using XF24 Analyzers equilibrated within the sealed chamber at ∼1% O_2_) (**B**). MCF10A and MB231 cells were exposed to ∼1% O_2_ for 0–5 h, and nuclear HIF-1α and cytoplasmic LDHA protein levels were assessed using Western blot analysis (representative image shown). Lamin A/C and β-actin were used as protein loading controls for nuclear and cytoplasmic fractions, respectively (**C**). Cells were exposed to atmospheric OT (closed bars) or ∼1% O_2_ (open bars) for 5 h, and the rate of 2-NBDG (0.3 mM) uptake, a fluorescence glucose analog, was measured. 2-NBDG fluorescence was normalized to protein levels (**D**). Values represent means ± SEM, n = 12. * p≤0.05 compared MCF10A. # p≤0.05 compared to cultures under normoxic conditions.

To determine the effects of decreasing *pO_2_* on OCR and ECAR, an XF24 extracellular flux analyzer (Seahorse Bioscience, North Billerica, MA) was placed in a controlled atmosphere chamber (PLAS Labs, Lansing, MI), and the chamber was equilibrated to 1% oxygen ±0.5% by flushing with argon gas (Fig. 1A). The pressure inside the controlled chamber was maintained at 760 mmHg and the temperature at ≤37°C. A second instrument was maintained at room air (∼20%, normoxia), and parallel plates were assayed and used as normoxic controls for each XF assay. Since the media surrounding the cells is equilibrated to room air prior to the start of the XF assay, an exponential loss of oxygen can be measured in the media as it equilibrates with the surrounding low oxygen atmosphere during hypoxia exposure (Fig. 1B). The KSV algorithm developed by Seahorse Bioscience was used to calculate OCR in all XF assays [27] with edge offset set to 1.0 and delta to 50 mmHg. Since the plate is equilibrated to normoxia, loss of oxygen from the measurement chamber due to reduced ambient oxygen was corrected by subtracting all experimental values by the OCR obtained in wells without cells. OCR and ECAR measurements were made every 8 min, and first three readings after the initiation of the assay were omitted from the calculation, as the instrument stabilizes during this period. OCR and ECAR traces were obtained over approximately 400 min in the hypoxia chamber and were fit using linear regression analysis. The slope of each fitted line is reported.

### Immunoblot Analysis

Nuclear and cytosolic fractions were isolated using NE-PER Nuclear and Cytoplasmic Extraction Reagents (Pierce, Rockford, IL). HIF-1α was detected with mouse anti-human HIF-1α antibodies (1∶2500; Transduction Laboratories, Lexington, KY) as previously described [28].

### Determination of Glucose Uptake

Glucose uptake was determined using a fluorescent nonhydrolyzable glucose analog, 2-NBDG. Cells exposed to normoxia or 5 h hypoxia were treated with 0.3 mM 2-NBDG, and 2-NBDG uptake was determined as previously described [28]. Fluorescence was measured using optical filters for fluorescein detection (excitation/emission maxima of 465/540 nm). The initial rate of 2-NBDG uptake was normalized to protein content measured by the Bradford method.

### Statistical Analysis

Data reported are means ± s.e.m. for n ≥3, as indicated in figure legends. Statistical significance was evaluated by one-way analysis of variance (ANOVA) among the groups using GraphPad Prism 4. The minimum level of significance was set at p<0.05. Tukey’s Multiple Comparisons test was used for post-hoc analysis of significance between groups.

## Results

### Validation of the Cellular Response to Low OT in XF24 Instrument

To measure the bioenergetic response of BCCs in response to OTs ranging from 20% to <1%, an XF24 Analyzer maintained in a controlled atmosphere chamber equilibrated to 1% oxygen was used as shown in Figure 1A. First, we determined whether our experimental protocol results in a prototypical response to low OT [13], [29]–[32]. Cells were maintained in media equilibrated to room air and loaded into the XF24 Analyzer. Over a period of 650 min, an exponential loss of O_2_ from the media surrounding the cells was consistently observed (Fig. 1B). Under these experimental conditions, viability of the cells was not affected (data not shown). Since HIF-1α is a hallmark of the cellular response to low OT, stabilization and nuclear translocation of HIF-1α was also determined from cells exposed to low OT in the XF24 instrument. Upon exposure to low oxygen (1% O_2_), levels of nuclear HIF-1α protein was rapidly increased within 1h in MB231 cells, while very little nuclear HIF-1α was detected in MCF10A cells at the same time point (Fig. 1C). Lactate dehydrogenase A (LDHA), a transcriptional target of HIF-1α [33], was also assessed after exposure to low OT, and LDHA levels increased in MB231 cells over the time period measured, but remained stable in MCF10A cells in response to low OT (Fig. 1C).

In addition, the initial rate of glucose uptake was monitored after exposure to atmospheric air or 1% O_2_ for 5 h. In MB231 cells exposed to 1% O_2_, glucose uptake was significantly increased (p≤0.001) when compared to cells maintained under normoxic conditions (Fig. 1D). In contrast, there was no significant difference between the rate of glucose uptake in MCF10A cells maintained at either normoxia compared to 1% O_2_ (Fig. 1D). These data indicate that MB231 cells exhibit a prototypical response towards low OT within 5 hours of being transferred from normoxia to 1% oxygen, and no appreciable response was detected in the non-tumorigenic cells.

### BCCs Enhance Glycolysis in Response to Decreasing OT

**Figure 2:**
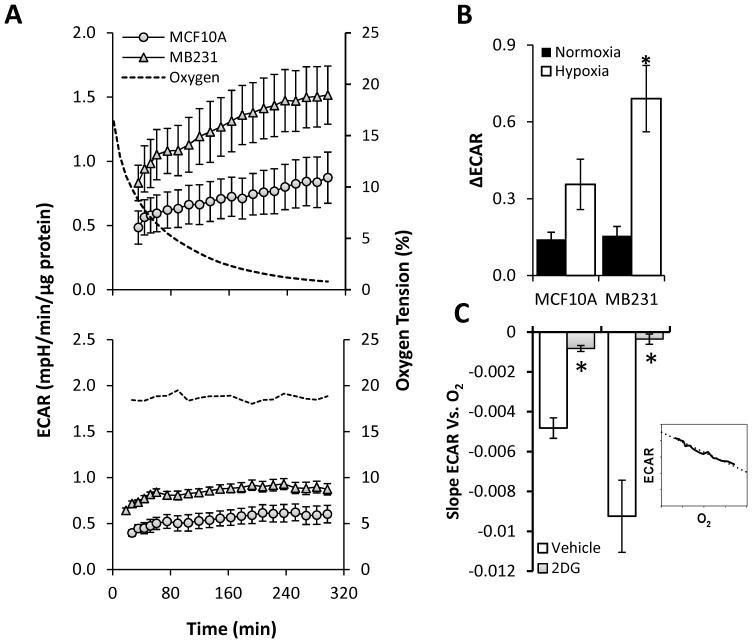
Effect of different OTs on extracellular acidification rate (ECAR) in breast epithelial and cancer cells. ECAR was determined in non-tumorigenic cells (MCF10A) and MB231over time in an atmosphere of reducing OT as described in Figure 1. A representative O_2_ trace (dotted line) during the course of the experiment is shown for reference. The change in ECAR was measured in MCF10A (circles) and MB231 (triangles) during reducing OT (**A upper panel**) and at atmospheric air (**A lower panel**). The change between the initial and the final ECAR under low OT and atmospheric air were determined (**B**). Exposure to reducing OT was performed in the absence (closed bars) or presence (open bars) of 2-deoxy-D-glucose (20 mM). Linear regression analysis was performed on each sample (see inset), and the average slope for each condition is shown (**C**). Values represent means ± SEM, n = 5–15. * p≤0.05 compared to MCF10A. # p≤0.05 compared to the respective untreated control.

Once we established the experimental protocol for this study, we sought to examine the real-time response of BCCs and non-tumorigenic MCF10A cells on ECAR (an indicator of glycolytic rate) in real-time during the exposure to decreasing oxygen level. Under normoxic conditions, basal ECAR was significantly higher in MB231 cells (Fig. 2A lower panel) as well as in metastatic MCF10A clones (Figure S1B) when compared to MCF10A cells which is in agreement with Warburg effect [34]. These values were relatively stable under normoxia over the course of the experiment in MB231 (Fig. 2A lower panel) and MCF10CA clones (Figure S1B). However, as shown in Figure 2A upper panel, ECAR, which was already higher under normoxic conditions, was further stimulated in the MB231 cells and metastatic MCF10CA clones (Figure S1A) as the oxygen tension (plotted as a dotted line) decreased over time. In contrast, the ECAR of MCF10A cells did not significantly change during the course of the experiment. The difference between the initial and final ECAR (ΔECAR) in MB231 was increased by 6-fold (Fig. 2B) and that of MCF10CA clones ranged from 5 to 11-fold (Figure S1C) when compared with normoxia. However, this difference was not significant in MCF10A cells (Fig. 2B). A plot of ECAR responses as a function of OTs revealed a strong inverse correlation between ECAR and OT for MB231 and metastatic MCF10CA clones (Figure S1D), and the slope of the line was significantly higher (p≤0.05) for MB231 (Fig. 2C and metastatic MCF10CA clones (Figure S1E; open bars) when compared to MCF10A cells, indicating BCCs respond to decreasing oxygen levels by proportionally increasing their basal glycolytic rate.

To confirm that the increase in ECAR is indeed due to increased glycolysis, ECAR during the exposure to decreasing oxygen levels was assessed in the presence of 2DG (20 mM), a glucose analog which cannot be metabolized to lactate through glycolysis [35]. Cells were pre-incubated in 2DG-containing running media for 1 h prior to the beginning of the XF24 assay. 2DG treatment alone caused a significant decrease in basal ECAR in MB231, but not in the MCF10A cells most likely due to the fact that BCCs rely more on glycolysis under basal conditions than their normal counterparts. Moreover, upon exposure to gradual decrease in OT in the presence of 2DG, the stimulation of ECAR was completely blocked in MB231 (Fig. 2C; gray bars) as well as in metastatic MCF10CA clones (Figure. S1E; grey bars). Together, these results indicate that stimulation of ECAR in response to low OT is the direct result of increased glycolytic flux.

### Changes in Oxygen Consumption in BCCs Under Decreasing OT

**Figure 3:**
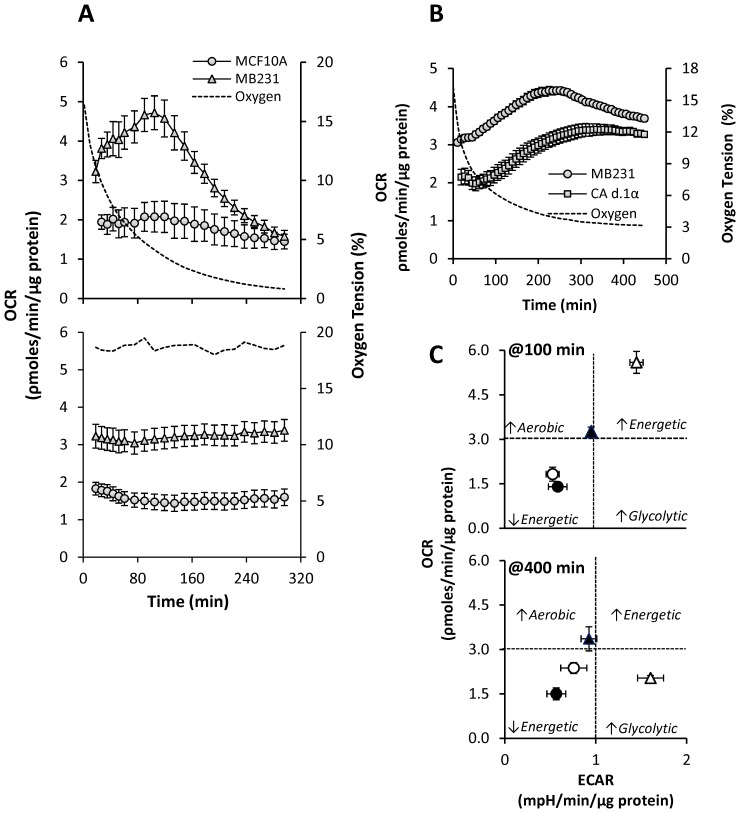
Effect of reducing OT on oxygen consumption rate (OCR) in breast epithelial and cancer cells. OCR was determined in non-tumorigenic (MCF10A) and MB231 over time during reducing OT as described in Figure 1. A representative O_2_ trace (dotted line) during the course of the experiment is shown for reference. OCR was measured over time in MCF10A (circles), and MB231 (triangles) (**A upper panel**)**.** OCR measured over time equilibrated at atmospheric air (**A lower panel**). OCR traces of MB231(circles) and MCF10CA d1.α at stable 4% OT are shown (**B**). The bioenergetic state of MCF10A (circles) and MB231 (triangles) cells was determined from data in Figures 2 and 3 at atmospheric air OT (closed) or at low OT (open) at 100 (**C upper panel**) and 300 (**C lower panel**) min by constructing a 2D plot of OCR versus ECAR.

Having established that our XF assay protocol results in a prototypical glycolytic response to low OT, we next investigated the changes in mitochondrial oxygen consumption under the same conditions. Surprisingly, we observed that as OT initially decreases (21% to approximately 4%), surprisingly, OCR was stimulated in the MB231. Then, as OT continued to decrease further (approximately 4% to 1%), loss of oxygen results in loss of OCR (Fig. 3A upper panel). Metastatic MCF10CA clones also showed similar response in OCR as MB231 cells (Figure S2A). In MCF10A cells, we found a different response whereby no initial stimulation of OCR was observed, and only at very low OT a decrease in OCR was observed. This suggests that the mechanism of maintaining mitochondrial bioenergetics under low oxygen tension in normal breast epithelial and cancer cells is intrinsically different. Cells assayed in an instrument equilibrated to room air showed relatively stable OCR over the course of the experiment (Fig. 3A lower panel and Figure 2B).

Plotting OCR as a function of oxygen showed a biphasic response to decreasing OT in MB231 as well as MCF10CA clones which is not apparent in MCF10A cells (Figure S2C). To further confirm the stimulation of OCR at 4–5% oxygen, BCCs maintained at atmospheric OT were equilibrated to 4% O_2_ instead of 1% for 7 h (Fig. 3B). Here, both MB231 and MCF10CA.d1α cells stimulated and then maintained an elevated OCR at a steady level throughout the experimental period suggesting that initial increase in OCR due to low OT is not transient, and BCCs actively maintain an elevated OCR at specific OTs (4%).

Next, linear regression analysis of oxygen versus OCR was performed. Since the response was biphasic, the traces were fitted first from normoxia to the peak OCR (∼4% oxygen) depending on the cell line (see inset Figure S2D), and then fitted from the peak OCR to the lowest oxygen level (<1% oxygen, see inset Figure S2E). Data from MCF10A cells were fit using the oxygen levels which represented the peak for each of the breast cancer cell lines, and there was no significant difference between the calculated slopes (data not shown). The slopes obtained for the early stimulation phase of OCR, which were significantly larger (p≤0.01) for the BCCs when compared to MCF10A cells (Figure S2D). Additionally, there is a stronger correlation between oxygen and OCR in this phase for the BCCs (R^2^ = 0.89, 0.96, 0.98 for MB231, 10CA a.1, and 10CA d.1α, respectively) than in MCF10A cells (R^2^ = 0.01).

The declining phase of OCR was also analyzed in the same manner. Again, significantly larger slopes (p≤0.001) were calculated for the BCCs when compared to MCF10A cells (Figure S2E); however, there was no longer a difference in the correlation between oxygen and OCR between breast cancer and non-tumorigenic cells (R^2^ = 0.99, 0.96, 0.98, 0.97 for MB231, 10CA a.1, 10CA d.1α, and MCF10A, respectively). These data demonstrate that the early simulation of OCR in response to decreasing oxygen is a phenomenon unique to BCCs compared to non-tumorigenic cells. However, this inverse relationship of OCR to oxygen is no longer apparent in the cancer cells at lower oxygen levels likely because oxygen becomes limiting in all cells examined.

### Bioenergetics of BCCs under Decreasing OT

The effect of different OTs on mitochondrial bioenergetics of BCCs and MCF10A may be represented as a plot of OCR vs. ECAR and representative plots at 100min and 300 min are shown in Figure 3C (upper panel). The effect on bioenergetics in MB231 (Fig. 3C) were pronounced when OTs reached between 4–5% at ∼100 min. (Fig. 3C upper panel). MB231 cells became more energetic (higher OCR and ECAR) state during this time point (open symbols) when compared with normoxia (closed symbols). On the other hand, at 400 min, where oxygen limits mitochondrial respiration, MB231 cells (Fig. 3C lower panel) were highly glycolytic in response to hypoxia (open symbols) and exhibited a relatively low level of aerobic metabolism. However, MCF10A cells did not show any bioenergetic changes and were relatively less energetic at all oxygen tensions.

### Increased Oxygen Consumption in BCCs Occurs through Mitochondrial Respiration

**Figure 4:**
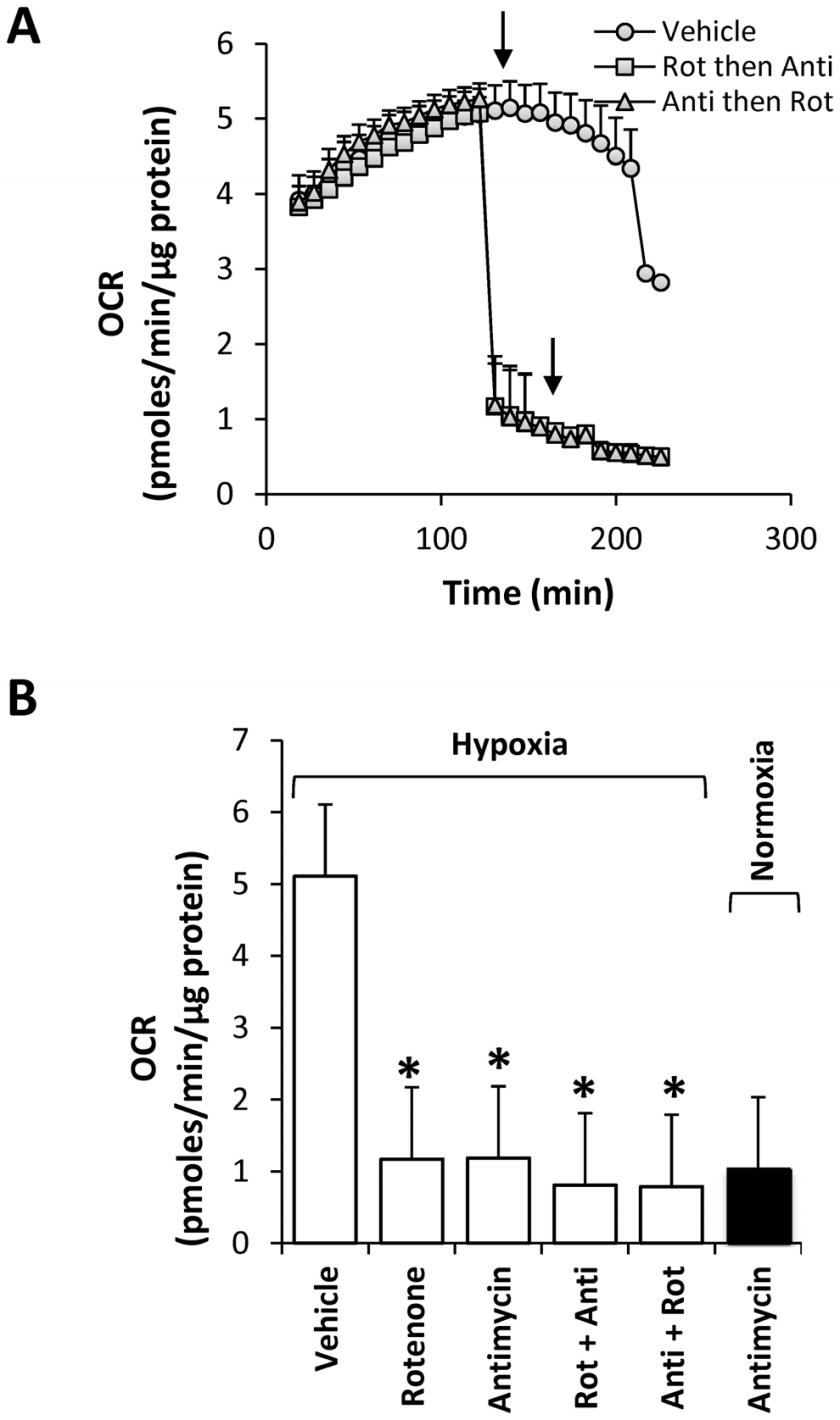
Effect of mitochondrial inhibitors on increased OCR in MB231 cells during hypoxia. At peak OCR, MB231 cells were treated with vehicle alone (ethanol/DMSO; closed circles), or treated sequentially (Inhibitor 1 then Inhibitor 2), with the Complex I inhibitor rotenone (0.5 µM) followed by the complex III inhibitor antimycin A (10 µM) (open squares), or antimycin A (10 µM) followed by rotenone (0.5 µM) (open triangles) at the times indicated by the arrows. Comparison of OCRs approximately 10 min after addition of inhibitor 1 (either Rotenone or Antimycin), and approximately 20 min after the addition of inhibitor 2 (Rotenone+Antimycin, or Antimycin+Rotenone) (**B**). For comparison, antimycin A (10 µM) was added to MB231 cells maintained under normoxia for 105 min and OCR was determined approximately 10 min after antimycin addition. Values represent means ± SEM, n = 5–10; *p<0.01 compared to vehicle.

In order to identify the site at which the increased oxygen consumption occurs during reducing OT, we initially tested whether mitochondria is involved in this process. To test this, MB231 cells were treated at peak OCR with either the Complex I inhibitor rotenone or the Complex III inhibitor antimycin A alone or one followed by the other (Fig. 4A). All the treatments at peak OCR completely inhibited the biphasic response in MB231 cells. Moreover, the observed inhibition occurred to a similar extent as seen by antimycin A under normoxia (Fig. 4B). To further confirm that the source of oxygen consumption is mitochondrial, a number of other inhibitors such as oxypurinol (xanthine oxidase inhibitor), catalase (H_2_O_2_ scavenger), L-N^G^-nitroarginine methyl ester (L-NAME; nitric oxide synthase inhibitor), MnTMPyP (superoxide scavenger), chloramphenicol (cytochrome P450 inhibitor), and nicotinamide nucleotides (cell surface NADH oxidase inhibitor) were tested. In these studies, none of the compounds inhibited the biphasic response in MB231 during the exposure to decreasing OT (Figure S3). Together, these data suggest that the increase in oxygen consumption which occurs in the early phase is primarily due to increased oxygen consumption by the mitochondrial electron transport chain, and this result is unique to BCCs compared to MCF10A cells.

### Loss of HIF-1α Completely Abolishes the Biphasic Response in OCR and Increase in ECAR in BCCs

**Figure 5:**
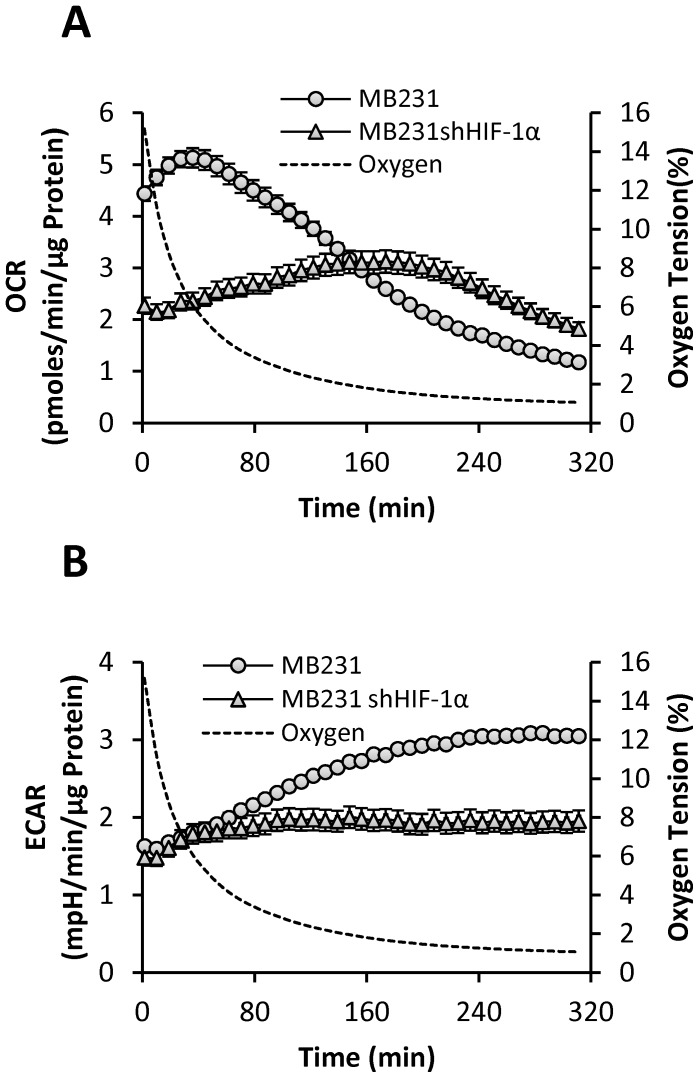
Effect of different OTs in MB231 and MB231^shHIF-1α^ cells on OCR and ECAR. MB231 cells (circles) or MB231 cells deficient in HIF-1α (triangles) were exposed to reducing OT as described in Figure 1. OCR (**A**) and ECAR (**B**) were simultaneously measured over time.

MB231 cells are highly metastatic and have high basal levels of HIF-1α protein, while MCF10A cells lack HIF-1α under normoxic conditions. Therefore we hypothesized that HIF-1α might play a central role in modulating the cancer cell bioenergetics in response to reducing OT. In order to define the mechanism of biphasic response in OCR and increasing ECAR during hypoxic exposure, we generated HIF-1α deficient MB231 (MB231 ^shHIF-1α^) cells. MB231 and MB231 ^shHIF-1α^ cells were exposed to reducing OTs from atmospheric air to <1% O_2_ as described above, and OCR and ECAR were measured simultaneously (Fig. 5). Our results show that knockdown of HIF-1α significantly reduced the ability of HIF-1α deficient MB231 cells to respond bioenergetically to reducing OT (Fig. 5A). More interestingly, loss of HIF-1α function completely abolished the biphasic response in OCR in MB231 ^shHIF-1α^ cells. Although there was no significant difference in basal ECAR, loss of HIF-1α also completely prevented steady increase of ECAR in MB231 ^shHIF-1α^ compared to MB231 cells (Fig. 5B). These observations suggest that HIF1α is responsible for the maintenance of basal OCR under atmospheric OT, as well as alterations in ECAR and OCR in response to changing OT. These findings also provide evidence for the direct crosstalk between mitochondria and HIF-1α in the metabolic phenotype of breast cancer cells.

### BCCs Require HIF-1α to Restore and Maintain OCR both during De-oxygenation and Re-oxygenation

**Figure 6:**
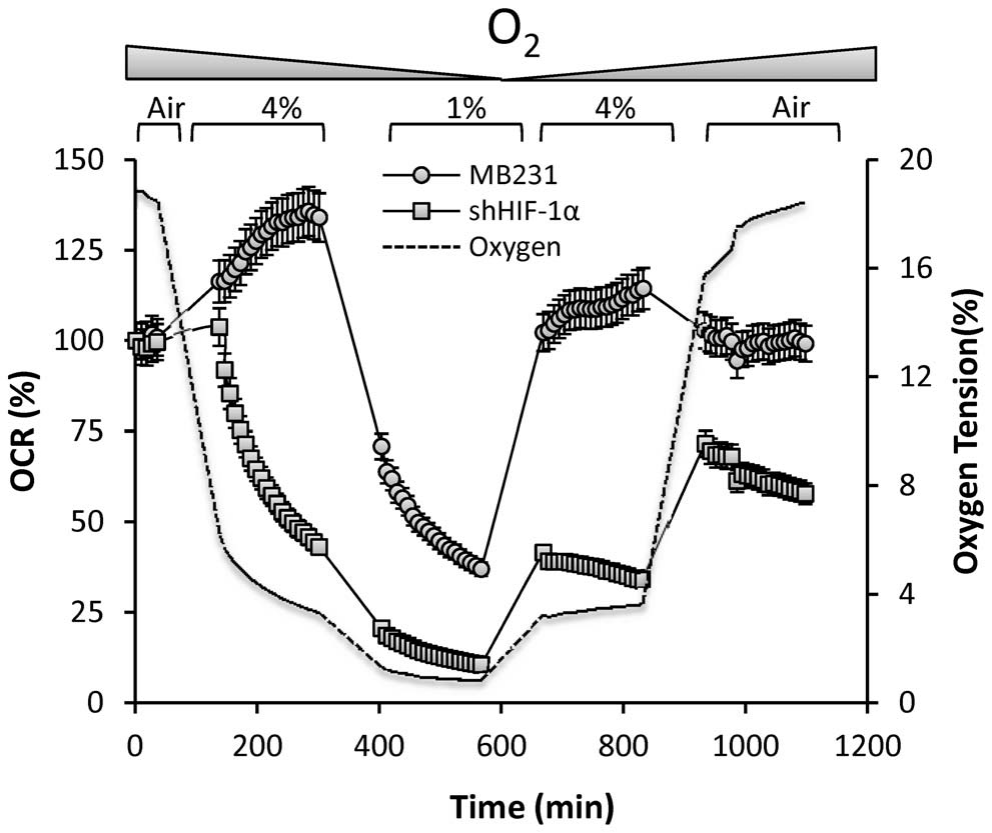
Determination of the bioenergetic flexibility of MB231 and MB231^shHIF-1α^ cells during de-oxygenation and re-oxygenation MB231 (circles) and MB231^ shHIF-1α^ (square) cells were equilibrated to atmospheric OT and is de-oxygenated to 4% and then to 1%. The chamber is re-oxygenated step-wise to 4% and then back to atmospheric OT. Several OCR measurements were made at each step and were plotted over time. A representative O_2_ trace (dotted line) during the course of the experiment is shown for reference.

We sought to determine whether BCCs have highly flexible bioenergetics which can respond to dynamic fluctuations in oxygen, as would occur during intermittent tumor hypoxia. To this end, atmospheric air was de-oxygenated and maintained at 4% O_2_ and then decreased to 1%. This was followed by re-oxygenation back to 4% and then to atmospheric air. During this period several OCR measurements were made at each OT (Fig. 6). Our results demonstrate that as the OT reached 4%, the OCR increased to a peak of 134% of the basal rate (Fig. 6) and was stable at 4% O_2_ in both MB231 (Fig. 6) and MCF10CA.d1α cells (Figure S4). As the OT was lowered to 1%, a condition in which the O_2_ limits mitochondrial respiration, the OCR gradually decreased and was maintained at ∼39% of the basal rate. Interestingly, as the chamber was re-oxygenated to 4%, O_2_, MB231 cells again stimulated OCR above the initial basal rate (113% of basal). When the cells were equilibrated back to atmospheric air, the OCR was restored to the basal levels. In striking contrast, MB231 ^shHIF-1α^ cells rapidly down-regulated OCR to 46% and 11% of the basal rate at 4% and 1% O_2_ respectively. Moreover, these cells failed to restore the OCR to basel levels upon reoxygenation. These results demonstrate the dynamic regulation of mitochondrial function controlled by HIF-1α in response to OT fluctuations. This suggests that the crosstalk between HIF-1α and mitochondria plays a prominent role in restoring and maintaining mitochondrial bioenergetics when BCCs are exposed to intermittent hypoxia in the tumor microenvironment as well as during invasion and metastasis.

## Discussion

In the present study, we used real-time extracellular flux analysis to define the differential bioenergetic responses of non-tumorigenic breast epithelial cells and BCCs to alterations in oxygen tension. To this end, normoxic culture media was allowed to equilibrate with the atmosphere in a hypoxic chamber maintained at 1% oxygen (Fig. 1). We found that MB231 cells demonstrated prototypical HIF-1α-dependent responses to low OT. In contrast, MCF10A cells did not appreciably stabilize HIF-1α, increase LDHA, or stimulate glucose uptake in response to low OT. This was somewhat unexpected given that numerous cell types have been previously reported to participate in adaptation to low OT. For example, up-regulation of the glucose transporter-1 (Glut-1) has been observed in nearly all cell types examined. However, our results clearly indicate that BCCs, but not non-tumorigenic breast epithelial cells, respond rapidly to a low OT. This discrepancy may be due to the differences in the duration of exposure of MCF10A cells to low OT compared to other published reports.

We next examined the temporal changes in ECAR in these cells. BCCs exhibited high rates of extracellular acidification at normoxia relative to MCF10A cells (Fig. 2). This “aerobic glycolysis” is consistent with the familiar view elaborated by Otto Warburg that the basal rate of glycolysis of tumorigenic cells is significantly higher than their non-tumorigenic counterparts under normoxia [34]. Loss of HIF-1α completely blocked the increase in ECAR in response to reducing OT. Increased glycolysis in tumor cells in response to low OT has been proposed as an adaptive feature to incorporate nutrients into biomass needed to support rapid proliferation [36] and to maintain redox status [37]. In this study, we report for the first time that the ECAR in tumor cells increases linearly as oxygen decreases. In the tumor microenvironment, the pH is controlled by multiple processes including increased expression and activity of enzymes such as Na^+^/H^+^ exchangers, proton pumps, mono-carboxylate transporters, HCO_3_^–^ transporters, and membrane-bound and cytosolic carbonic anhydrases [29]. In addition, non-glycolytic reactions catalyzed by alanine transaminase and malic enzyme may also contribute to the formation of lactate [38]. However, here we confirmed that increased ECAR in cancer cells is the result of increased glycolysis since pretreatment of cells with 2DG decreased basal ECAR and completely attenuated the stimulation of ECAR in response to hypoxia.

We have shown clearly that BCCs under atmospheric OT have higher oxygen consumption than MCF10A cells suggesting that these BCCs are not only highly glycolytic, but also have highly active mitochondrial metabolism. These results are consistent with current thinking that many cancers are metabolically flexible and can derive energy substrates and cellular building blocks through multiple metabolic pathways [38]. Similar findings have also been reported for other breast cancer cell lines such as MCF-7 [39] and mammary xenografts [40].

Intriguingly, we observed a biphasic change in OCR in all BCCs studied in response to decreasing OT (Fig. 3). However, this effect was not exhibited by MCF10A cells suggesting the mitochondrial response to low OT is intrinsically different in non-tumorigenic versus tumorigenic cells. We showed that the increase in oxygen consumption during the early phase is due to increased mitochondrial oxygen consumption (Fig. 4). None of the non-mitochondrial sources of oxygen consumption are involved in mediating the biphasic response in OCR. Moreover, this early phase increase in OCR is apparently mediated by HIF-1α, as silencing of HIF-1α gene inhibited basal OCR at atmospheric OT and completely abolished biphasic response in OCR as well as the increase in ECAR under decreasing OT. Our findings also show that HIF-1α is essential for BCCs to maintain the bioenergetics and for the active regulation of the mitochondrial bioenergetics by sensing the environmental OT and helps to reach and maintain the attainable mitochondria function (Fig. 6) at any particular OT, a novel finding not previously reported. Such a response suggests that BCCs are highly sensitive to changes in oxygen and contain a significant metabolic reserve, both mitochondrial and glycolytic, compared to their normal counterparts. This HIF-1α-dependent bioenergetic flexibility may confer an adaptive advantage to cancer cells as they are exposed to the ever-changing landscape of tumor microenvironment [41]. For example, during the metastatic process, cells migrate away from the primary tumor and must survive transport to secondary sites and the new microenvironments therein.

At present we do not know how HIF-1α regulates precisely the bioenergetics of BCCs during the gradual decrease in OT or re-oxygenation. The observed changes in mitochondrial function may be at the level of substrate availability/accumulation within the mitochondrion or through changes in the specific activity of mitochondrial enzymes such as electron transport chain complexes. Previous reports show that HIF-1α activates pyruvate dehydrogenase kinase (PDK)-1 under hypoxic conditions that regulates mitochondrial oxygen consumption in cancer cells by inhibiting pyruvate dehydrogenase, the enzyme responsible for converting pyruvate into acetyl CoA [18], [42]. HIF-1α also controls mitochondrial respiration during hypoxia by exchanging subunit 4-1 of cytochrome c oxidase (COX) for the more efficient COX4-2 isoform [43]. This is a possible mechanism, as these studies were performed under long-term chronic hypoxic conditions sufficient to cause gene expression changes. Here, we have used a highly sensitive method to measure OCR (picomolar levels) and ECAR (mpH levels) which makes the direct comparison with previous studies more difficult. At present, we do not exclude the possible role of PDK1 and COX-4 subunit switching for the rapid bioenergetic adaptation of BCCs under reducing OT as these cells already have high levels of cellular HIF-1α under normoxic conditions. Nevertheless, all the BCC lines we tested showed similar response suggesting that there is a common HIF-1α-dependent pathway for BCCs to precisely sense specific OTs and regulate and maintain the bioenergetics which is not readily activated in normal epithelial cells.

Taken together, our results validate a novel technique to assess bioenergetics parameters during adaption to low OT in real-time. Moreover, we found that changes in metabolic pathways during low OT are fundamentally different in BCCs compared with non-tumorigenic breast epithelial cells specifically that BCCs rapidly increase glycolysis and transiently increase mitochondrial oxygen consumption as oxygen levels decrease which is exclusively mediated by HIF-1α. This experimental approach may be useful for the metabolic phenotyping of metastatic cancer cell lines or cells isolated from cancer patients irrespective of their site of origin or stage of tumor progression. This information may be translated into the clinics for the diagnosis and to take appropriate treatment decisions, evaluate treatment responses and its outcomes. It is worth mentioning that cancer associated fibroblasts and macrophages are exposed to low oxygen tension within the tumor mass like cancer cells and play predominant role in tumor progression. Currently we do not know the bioenergetics of these cell types, however, it is possible that these cells may respond differently compared to tumor or non-tumor epithelial cells and may have a different bioenergetic profile under low OT. These bioenergetic differences may be exploited in order to sensitize hypoxic, aggressive and treatment resistant tumor cells to apoptosis *in vivo*. Thus, our findings highlight cancer-specific metabolic pathways which may be targeted therapeutically, and describe a platform which may be utilized to screen compounds to this end Therefore, additional studies are warranted to further define the cellular events which drive the metabolic responses to low OT.

## Supporting Information

Figure S1 **Effect of different OTs on extracellular acidification rate (ECAR) in metastatic MCF10A clones.** ECAR was determined in metastatic MCF10A clones over time in an atmosphere of reducing OT as described in Figure 1. A representative O_2_ trace (dotted line) during the course of the experiment is shown for reference. The ECAR was measured in MCF10CA a.1 (circles) and MCF10CA d.1α (triangles) during reducing OT **(A**) and at atmospheric air (**B**). ECAR traces plotted against reducing OTs (**C**). The change between the initial and the final ECAR under low OT and atmospheric air were determined **(D)**. Exposure to reducing OT was performed in the absence (closed bars) or presence (open bars) of 2-deoxy-D-glucose (20 mM). Linear regression analysis was performed on each sample (see inset), and the average slope for each condition is shown **(E)**. Values represent means ± SEM, n = 5–15. * p≤0.05 compared to MCF10A. # p≤0.05 compared to the respective untreated control.
(TIF)

Figure S2 **Effect of reducing OT on oxygen consumption rate (OCR) in metastatic MCF10A clones.** OCR was determined in MCF10A clones over time during reducing OT as described in Figure 1. A representative O_2_ trace (dotted line) during the course of the experiment is shown for reference. OCR was measured over time in MCF10CA a.1 (circles) and MCF10CA d.1α (triangles) **(A)**. OCR measured over time equilibrated at atmospheric air (**B**). OCR traces of MCF10A (closed circles), MB231 (closed triangles), MCF10CA a.1 (grey circle) and MCF10CA d.1α (gray triangles) were plotted against reducing OTs (**C**). Linear regression analysis was performed on each sample (see insets) from ‘normoxia’ to the peak response of OCR (**D**) and from the peak response of OCR to ‘hypoxia’ (**E**). The average slope for each cell line is shown. Values represent means ± SEM, n = 10–15. * p≤0.05 compared to MCF10A.
(TIF)

Figure S3 **Effect of antioxidants on hypoxia-induced OCR in breast cancer cells.** Experimental setup and conditions were same as described for Figure 2 except the cells were pre-treated with antioxidants for 1 h prior to hypoxia exposure and OCR measurement. Cells were pretreated with catalase (100 U), L-NAME (100 µM), NADPH (1 mM), NADH (1 mM), Oxypurinol (100 µM), Chloramphenicol (300 µM), or MnTMPyP (10 µM, 50 µM, or 100 µM). Values represent mean relative change in OCR at 100 min after hypoxia exposure compared with vehicle alone ± SEM, n = 6–12.
(TIF)

Figure S4 **Determination of the bioenergetic flexibility of MCF10CA d.1α during de-oxygenation and re-oxygenation.** MCF10CA d.1α cells (triangles) were equilibrated to atmospheric OT and is de-oxygenated to 4% and then to 1%. The chamber is re-oxygenated step-wise to 4% and then back to atmospheric OT. Several OCR measurements were made at each step and were plotted over time. A representative O_2_ trace (dotted line) during the course of the experiment is shown for reference.
(TIF)
