# Supplementary figures and images for: Mitochondrial Bioenergetics of Metastatic Breast Cancer Cells in Response to Dynamic Changes in Oxygen Tension: Effects of HIF-1α
Source: PLoS One. 2013 Jun 28;8(6):e68348. doi: 10.1371/journal.pone.0068348 (PMC3696014; doi:10.1371/journal.pone.0068348)

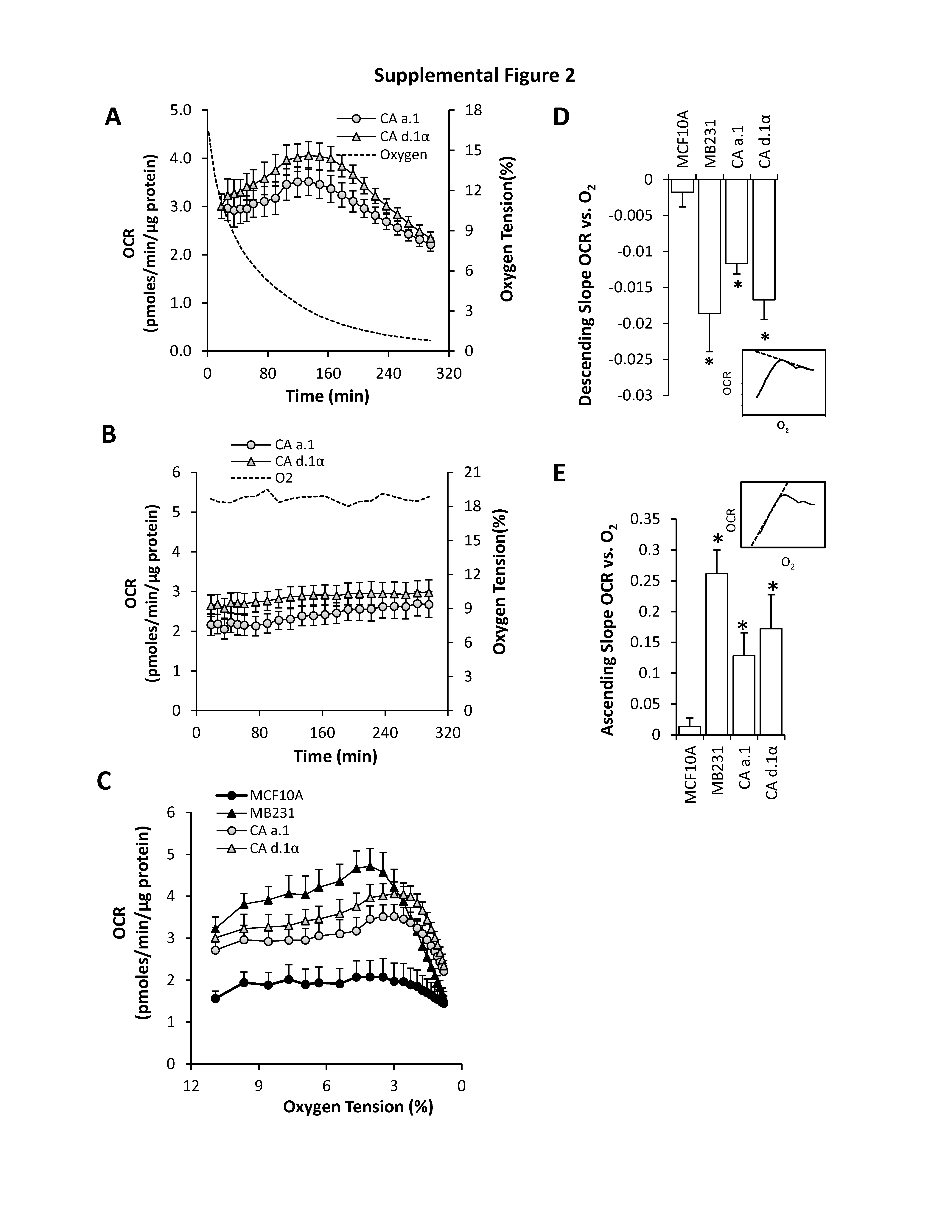

Supplement: Figure S2 — Effect of reducing OT on oxygen consumption rate (OCR) in metastatic MCF10A clones. OCR was determined in MCF10A clones over time during reducing OT as described in Figure 1. A representative O2 trace (dotted line) during the course of the experiment is shown for reference. OCR was measured over time in MCF10CA a.1 (circles) and MCF10CA d.1α (triangles) (A). OCR measured over time equilibrated at atmospheric air (B). OCR traces of MCF10A (closed circles), MB231 (closed triangles), MCF10CA a.1 (grey circle) and MCF10CA d.1α (gray triangles) were plotted against reducing OTs (C). Linear regression analysis was performed on each sample (see insets) from ‘normoxia’ to the peak response of OCR (D) and from the peak response of OCR to ‘hypoxia’ (E). The average slope for each cell line is shown. Values represent means ± SEM, n = 10–15. * p≤0.05 compared to MCF10A. (TIF) [file pone.0068348.s002.tif]

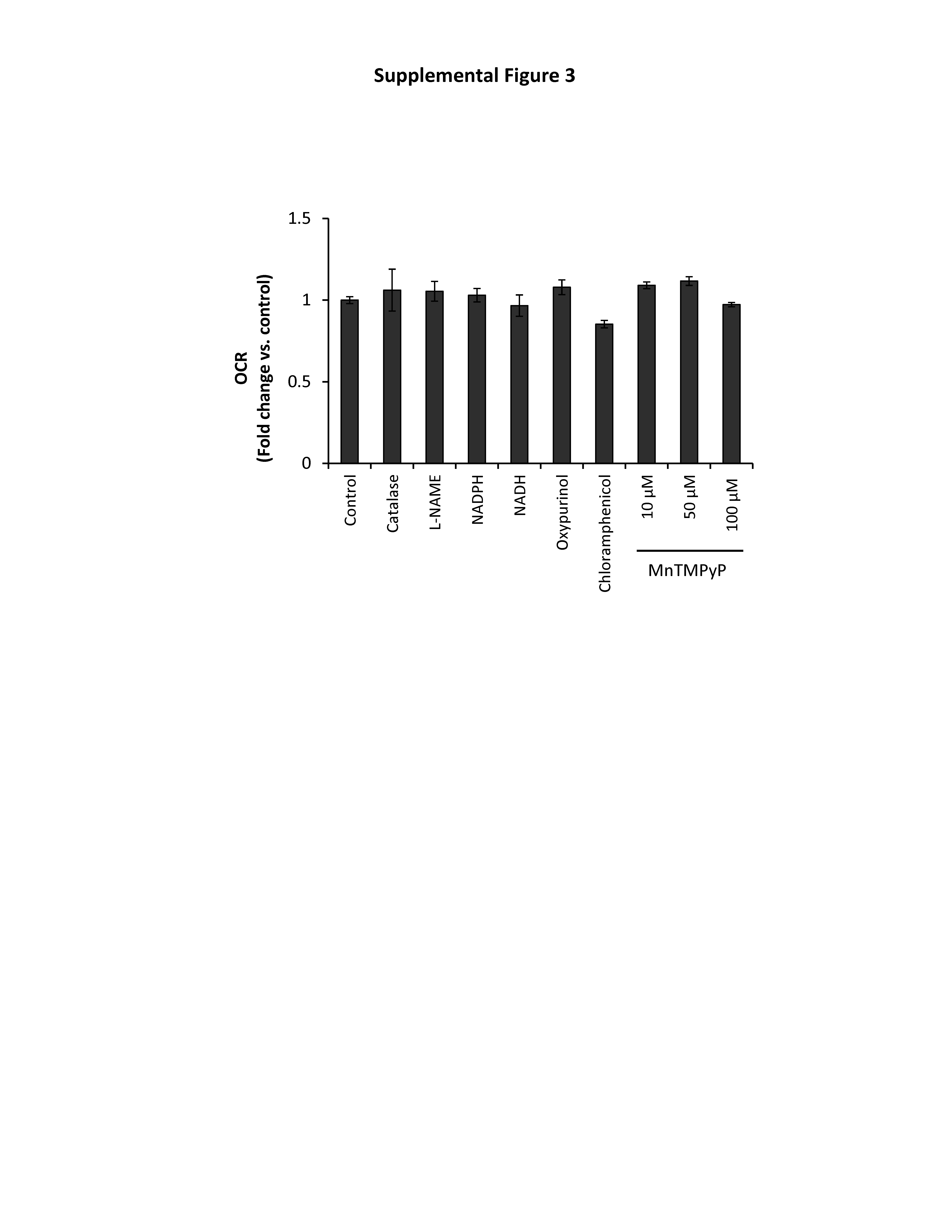

Supplement: Figure S3 — Effect of antioxidants on hypoxia-induced OCR in breast cancer cells. Experimental setup and conditions were same as described for Figure 2 except the cells were pre-treated with antioxidants for 1 h prior to hypoxia exposure and OCR measurement. Cells were pretreated with catalase (100 U), L-NAME (100 µM), NADPH (1 mM), NADH (1 mM), Oxypurinol (100 µM), Chloramphenicol (300 µM), or MnTMPyP (10 µM, 50 µM, or 100 µM). Values represent mean relative change in OCR at 100 min after hypoxia exposure compared with vehicle alone ± SEM, n = 6–12. (TIF) [file pone.0068348.s003.tif]
